# Analysis of nectar from low‐volume flowers: A comparison of collection methods for free amino acids

**DOI:** 10.1111/2041-210X.12928

**Published:** 2017-11-14

**Authors:** Eileen F. Power, Daniel Stabler, Anne M. Borland, Jeremy Barnes, Geraldine A. Wright

**Affiliations:** ^1^ Institute of Neuroscience Newcastle University Newcastle upon Tyne UK; ^2^ Botany Department School of Natural Sciences Trinity College Dublin Dublin 2 Ireland; ^3^ School of Natural and Environmental Science: Biology Newcastle University Newcastle upon Tyne UK

**Keywords:** amino acid, *Calluna vulgaris*, nectar, pollinator, sampling method, UHPLC

## Abstract

1. Floral nectar is a reward offered by flowering plants to visiting pollinators. Nectar chemistry is important for understanding plant nutrient allocation and plant–pollinator interactions. However, many plant species are difficult to sample as their flowers are small and produce low amounts of nectar.
2. We compared the effects of different methods of nectar collection on the amino acid composition of flowers with low volumes of nectar. We used five methods to collect nectar from 60 (5 × 12) *Calluna vulgaris* flowers: microcapillary tubes, a low‐volume flower rinse (the micro‐rinse method, using 2 μl water), filter paper, a high‐volume flower rinse (2 ml water) and a flower wash (2 ml water). We analysed the samples for free amino acids using quantitative UHPLC methods .
3. We found that the micro‐rinse method (rinsing the nectary with enough water to only cover the nectary) recovered amino acid proportions similar to raw nectar extracted using microcapillary tubes. The filter paper, 2 ml rinse and 2 ml wash methods measured significantly higher values of free amino acids and also altered the profile of amino acids. We discuss our concerns about the increased contamination risk of the filter paper and high‐volume rinse and wash samples from dried nectar across the floral tissue (nectar unavailable to floral visitors), pollen, vascular fluid and cellular fluid.
4. Our study will enable researchers to make informed decisions about nectar collection methods depending on their intended chemical analysis. These methods of sampling will enable researchers to examine a larger array of plant species' flowers to include those with low volumes of nectar.

## 1 INTRODUCTION

Flowering plants produce floral nectar to attract pollinating animals. The caloric value of the food pollinators receive when visiting flowers affects pollinator visitation (Chittka & Schurkens, 2001) and hence plant–pollinator co‐evolution and community structure (Price, 1997). Nectar is the main source of carbohydrates for pollinators and is composed of water and simple sugars (sucrose, glucose and fructose) ranging from 7% to 70% w/w (Nicolson, Nepi, & Pacini, 2007). Although 1,000–100,000 times less concentrated in nectar than sugars (Gottsberger, Schrauwen, & Linskens, 1984), free amino acids are the second‐most abundant nectar solutes (Petanidou, Van Laere, Ellis, & Smets, 2006). For pollinators that cannot eat pollen such as adult Lepidopterans, nectar is an important source of dietary amino acids (Baker, 1977; Baker & Baker, 1977) that may have a profound effect on longevity and influence pollinator behaviour (Gardener & Gillman, 2002; Hendriksma, Oxman, & Shafir, 2014; Inouye & Waller, 1984; Petanidou et al., 2006; Simcock, Gray, & Wright, 2014).

Studies directly linking the amino acid composition of nectar to pollinator behaviour have been few, in part because measuring amino acids in nectar is difficult and quantification requires specialized equipment (Nepi, 2014). Early studies by Baker and Baker (1973) quantified total amino acid content using simple ninhydrin staining techniques: a colorimetric method that revealed the presence of amines. Their subsequent studies used thin‐layer chromatography (TLC) to separate some amino acids and quantify them according to an ordinal scale (Baker and Baker, 1976, 1977, 1979; Baker, Opler, & Baker, 1978). However, precise quantification and identification of amino acids require the use of advanced chromatography methods such as high‐performance liquid chromatography (HPLC). Most published methods for amino acid analysis require a derivatization step prior to HPLC analysis, which may lead to sample loss and make it difficult to analyse flowers with very low volumes of nectar.

Most published studies of amino acids in nectar have used one of two techniques for collecting nectar: glass capillary tubes (Gardener and Gillman, 2001; Gottsberger, Arnold, & Linskens, 1990) or filter paper wicks (Petanidou et al., 2006). Glass capillary tubes are a good way to collect nectar, but they require sample volumes >0.5 μl and may not recover all nectar found within a flower. The nectar volume of many plant species, especially those pollinated by bees or other insects, is often <0.5 μl per floret. For this reason, ecologists studying nectar resort to other methods, such as filter paper wicks to collect nectar (Kearns & Inouye, 1993; McKenna & Thomson, 1988; Petanidou et al., 2006). One study compared the carbohydrates rendered by four different techniques for collecting nectar (microcapillary tubes, filter paper, washing and rinsing, see Table 1) (Morrant, Schumann, & Petit, 2009). These authors found that washing and rinsing returned higher estimations of the sugars available in nectar, and for this reason, they recommended washing and rinsing as methods for collecting from flowers with small nectar volumes (Morrant et al., 2009). While these methods work well for carbohydrates, no one has compared these techniques to identify how they could influence the amino acid profile of nectar studies. One foreseeable problem with nectar samples that have been collected by washing and rinsing is that these methods could overestimate the available amino acids if free amino acids from floral pollen were washed into the sample.

**Table 1:** Methods used to extract nectar from flowers and their suitability in relation to nectar volume (low (<1 μl) or high)

Method	Description	Nectar volume suitability	Selected references
Microcapillary tubes	Suction of raw nectar by capillary action up a narrow tube of known volume. Nectar volume can be quantified based on how much it has displaced the air inside the tube. Nectar can be expelled from the tube for analysis	Low–high	Corbet (2003), Morrant et al. (2009)
Filter paper	Soakage of nectar onto filter paper wicks which are subsequently immersed in water. The filter paper material is removed leaving a nectar–water solution for analysis	Low–high	Kearns and Inouye (1993), McKenna and Thomson (1988), Morrant et al. (2009)
Wash	Washing flower in sealed tube of a known volume of distilled water, followed by removal of the flower, leaving behind a nectar–water solution for analysis	Low–high	Grunfeld, Vincent, and Bagnara (1989), Morrant et al. (2009)
Rinse	Pouring a known volume of distilled water over the nectaries of a flower. The collected nectar–water solution can be used for analysis	Low–high	Nunez (1977), Mallick (2000), Morrant et al. (2009)
Micropipettes and microsyringes	Suction of raw nectar up a syringe or narrow tube, manually or with the aid of a pipette. Nectar volume can be quantified and analysed by expelling the nectar from the tube/syringe	High	Corbet (2003), Lanza, Smith, Suellen, and Cash (1995), Mallick (2000), Wykes (1952)
Centrifuge	The flower is secured in a centrifuge tube which is spun at high speed in a centrifuge to release nectar. Nectar is collected on the sides of the tube and can be removed using a microcapillary tube for quantification and analysis	High	Armstrong and Paton (1990), Swanson and Shuel (1950)
Aspirator	Nectar is drawn from the flower using a capillary tube (of known volume) attached to a power‐driven aspirator. Nectar volume can be quantified and analysed by expelling the nectar from the tube	High	Armstrong and Paton (1990)

Here, we report the first use of advanced UHPLC (ultra‐high‐performance liquid chromatography) with a specialized “in vial” derivatization step to compare and identify how different methods of nectar collection from flowers with small volumes of nectar affect the amino acid profile of nectar. We also report a new method—the “micro‐rinse” method—for collecting nectar samples for amino acid analysis from small‐volume flowers.

## 2 MATERIALS AND METHODS

### 2.1 Nectar collection

Five methods of nectar collection were compared for amino acid recovery using flowers collected from three plants of the same age of *Calluna vulgaris* L. (Ericaceae). To reduce variation based on flower age, plant and time of sampling (Nicolson et al., 2007), four flowers of similar maturity (open and showing no signs of senescence) were taken from each of three plants (*n* = 12 per method) at the same time for each collection method. Flowers were not bagged as they were not exposed to insect visitors in the laboratory (that may deplete resources). All nectar collection methods represent the standing crop (Corbet, 2003). Nectar was sampled from these flowers using the following methods: (1) microcapillary tubes (Corbet, 2003; Kearns & Inouye, 1993; McKenna & Thomson, 1988; Morrant et al., 2009); (2) filter paper wicks (Kearns & Inouye, 1993; McKenna & Thomson, 1988; Morrant et al., 2009); (3) washing in 2 ml of water (Morrant et al., 2009); (4) rinsing with 2 ml of water (Morrant et al., 2009); and (5) rinsing with 2 μl of water (micro‐rinse approach). The first four methods have been compared by Morrant et al. (2009) in terms of suitability for sugar recovery in low‐volume flowers, while we propose the fifth method as suitable for amino acid recovery. The details of each collection method are given below.

1. Microcapillary tubes (raw nectar): This method provides a means to estimate the volume of nectar obtained from individual flowers. In our experiments, we sampled nectar from 12 individual flowers using 1‐μl microcapillary tubes (Hirschmann Laborgeräte GmbH & Co. KG, Eberstadt, Germany). Nectar was drawn into the tubes by capillary action. This was performed with care to avoid damage to floral tissue and to prevent contamination with pollen grains. The volume of withdrawn nectar was quantified by measuring the length of the tube (mm) using a ruler and calculating the proportion of the tube that was filled with nectar. Each nectar sample was diluted with de‐ionized UHPLC gradient grade water (Fisher Scientific UK Ltd., Loughborough, United Kingdom) to meet minimal sample volume requirements for UHPLC analysis (UHPLC amino acid dilution: 10 μl requiring 1:65 dilution—see Sample preparation and analysis section).
2. Filter paper: Nectar was sampled from 12 flowers using filter paper wicks, adapted from Morrant et al. (2009). Twelve strips of Fisherbrand QL100 filter paper (Fisher Scientific UK Ltd., Loughborough, United Kingdom) with dimensions (5 × 42 mm, tapered to 1‐mm‐width tip at one end) were cut using sterile blades. Using sterile forceps, the edges of one filter paper tip were applied to the nectaries of one flower. Each strip was then placed in a sealed sterile vial (20 ml) containing 2 ml of de‐ionized UHPLC gradient grade water, soaked for 15 min and then agitated for 1 min.
3. Wash 2 ml: Nectar was sampled from 12 flowers using a washing method adapted from Morrant et al. (2009). Each flower was cut from the plant and placed in a sealed sterile vial (20 ml) containing 2 ml of de‐ionized UHPLC gradient grade water. The vial was agitated for 1 min.
4. Rinse 2 ml: Nectar was sampled from 12 flowers using a rinsing method adapted from Morrant et al. (2009). A flower was inverted over a 2‐ml sterile vial, and four successive rinses (0.5 ml) of de‐ionized UHPLC gradient grade water were expelled over the floral nectaries using a sterile pipette. It was not necessary to remove the flowers from the plant for this method.
5. Micro‐rinse: Nectar was sampled from 12 flowers using a novel rinsing method. Using a sterile pipette, 2 μl of de‐ionized UHPLC gradient grade water was expelled into a flower over the nectaries. The water was retained in the flower for 1 min, and then, the nectar–water solution was drawn into a 10‐μl microcapillary tube (Hirschmann Laborgeräte GmbH & Co. KG, Eberstadt, Germany) by capillary action. This was performed with care to avoid damage to floral tissue and prevent uptake of pollen grains into the sample. No floral tissue was removed prior to rinsing. The volume of withdrawn nectar–water solution was quantified by measuring the length of the tube (mm) using a ruler and calculating the proportion of the tube that was filled with solution. Each sample was diluted further with de‐ionized UHPLC gradient grade water to meet minimal sample volume requirements for UHPLC analysis (see above). It was not necessary to remove the flowers from the plant for this method. The 2 μl volume of water added to the nectary was chosen because it was sufficient to cover the nectary but not the anthers.
6. Filter paper control: To determine whether filter paper leaches amino acid contaminants into the nectar sample, ten filter paper wicks of similar type and dimensions to the above method were dipped using sterile forceps in ten sterile 2‐ml microcentrifuge tubes containing 1 μl of de‐ionized UHPLC gradient grade water. This procedure was designed to emulate nectar extraction from 12 flowers. Each strip was then placed in a sealed sterile vial (20 ml) containing 2 ml of de‐ionized UHPLC gradient grade water, soaked for 15 min and then agitated for 1 min.

When using filter paper, washing and rinsing 2 ml methods and the micro‐rinse method, it was necessary to obtain a separate estimate of the mean standing crop (nectar volume per flower) so that the mass of solutes in nectar per flower could be calculated. To obtain a standing crop value, the volume of nectar in 12 flowers was collected using 1‐μl capillary tubes. The mean volume recovered was 0.474 μl/flower (±0.06 *SE*); this value represents the mean amount of nectar present in the flower. For the washing and rinsing 2 ml and the filter paper methods, the amount of water used to rinse/wash (i.e. 2 ml) or to dilute (2 ml) was divided by the standing crop value.

For the micro‐rinse method, the mean recovery volume was 2.005 μl/flower (±0.110 *SE*) using a 10‐μl capillary tube. As we estimated the standing crop to be 0.474 μl/flower, a value of 2.005 μl/flower indicates that we did not recover all the nectar and all the rinse water from the flower. Given that the recovery volume was roughly equal to the volume we put into the flower, we reasoned that the amount of nectar possible to recover using this method was 1.53 μl (i.e. recovery volume—standing crop value). We subtracted the estimated recovery volume from the total volume collected using the micro‐rinse method to identify an estimate of the volume from each flower sampled. This was necessary to control for error in the volume estimation of the original sample; errors in this step with small volumes have dramatic effects on the calculation of the dilution factor (see Data S1).

Previous studies have used distilled water to dissolve nectar solutes from filter paper or wash and rinse nectar from flowers (Mallick, 2000; McKenna & Thomson, 1988; Morrant et al., 2009; Petanidou et al., 2006). We used de‐ionized UHPLC grade water which is free from amino acids and other ionic contamination (advanced HPLC grade water for HPLC gradient analysis, Fisher Sci, product no. 10221712). Samples were stored at −20°C for 1 week before UHPLC analysis.

### 2.2 Nectar sample preparation and analysis

#### 2.2.1 Filtration

For UHPLC amino acid analysis, 10 μl of sample was required. Some nectar collection methods produced enough sample volume for analysis (e.g. filter paper, wash 2 ml, rinse 2 ml methods and filter paper control). These samples were filtered using a sterile 0.45‐μm 4‐mm nylon Whatman Puradisc syringe filter to remove paper and plant material (note: filtering caused the loss of a significant amount of sample but enough remained for analysis). Low‐volume (<100 μl) samples were not filtered. Instead, they were diluted and centrifuged (see Section 2.2.2).

#### 2.2.2 Centrifugation

Microcapillary and micro‐rinse samples were diluted 65‐fold for amino acid analysis using de‐ionized UHPLC gradient grade water. These dilution factors were derived by diluting nectar so that amino acid concentrations matched those seen in the amino acid standards used to calibrate the chromatography instruments. Low‐volume samples were centrifuged for 10 min at 13,249* g* to separate soluble amino acids (supernatant) from any residual plant material.

#### 2.2.3 Amino acid analysis

UHPLC was used to measure concentrations of 21 amino acids: aspartic acid (asp), glutamic acid (glu), asparagine (asn), serine (ser), glutamine (gln), histidine (his), glycine (gly), threonine (thr), arginine (arg), alanine (ala), tyrosine (tyr) cysteine (cys), valine (val), methionine (met), gamma‐aminobutyric acid (GABA), tryptophan (trp), phenylalanine (phe), isoleucine (ile), leucine (leu), lysine (lys) and proline (pro) (listed in order of elution).

Using an automated pre‐column derivatization programme for the autosampler (Ultimate 3000 Autosampler, Dionex, Thermo Fisher Scientific Inc.), 10 μl of the diluted nectar was treated for 1 min with 15 μl of 7.5 mmol/L o‐phthaldialdehyde (OPA) and 225 mmol/L 3‐mercaptopropionic acid (MPA) in 0.1 M sodium tetraborate decahydrate (Na_2_B_4_O_7_·10 H_2_O), pH 10.2 and for 1 min with 10 μl of 96.6 mmol/L 9‐fluorenylmethoxycarbonyl chloride (FMOC) in 1 M acetonitrile. This was followed by the addition of 6 μl of 1 M acetic acid. After pre‐treating, 30 μl of the amino acid derivatives was then injected onto a 150 × 2.1 mm Accucore RP‐MS (Thermo Fisher Scientific Inc.) UHPLC‐column. Elution of the column occurred at the constant flow rate of 500 μl/min using a linear gradient of 3 to 57% (v/v) of solvent B over 14 min, followed by 100% solvent B for 2 min and a reduction to 97% solvent B for the remaining 4 min. Elution solvents were as follows: *A* = 10 mmol/L disodium hydrogen orthophosphate (Na_2_HPO_4_), 10 mmol/L Na_2_B_4_O_7_·10H_2_O, 0.5 mmol/L sodium azide (NaN_3_), adjusted to pH 7.8 with concentrated HCl and *B* = acetonitrile/methanol/water (45/45/10 v/v/v). The derivatives were detected via fluorescence (Ultimate 3000 RS Fluorescence Detector, Dionex, Thermo Fisher Scientific, OPA: excitation at 330 nm and emission at 450 nm, FMOC: excitation at 266 nm and emission at 305 nm) and quantified by automatic integration after calibration of the system with known amino acid standards. The instrument was calibrated twice per day by injecting amino acid standards (see example chromatogram, Figure S1) (which were comprised of a pre‐made solution of 17 amino acid standards for fluorescence detection (Sigma‐Aldrich). An additional four amino acids (available in solid form from Sigma‐Aldrich) were added to the solution for system calibration) with mean concentrations of 25 nmol/ml. The dual calibration every day was to ensure accuracy in peak identification given potential daily drift in amino acid elution times. Elution profiles were analysed using Chromeleon (Thermo Fisher Scientific Inc.), which automatically calculates solute concentrations (nmol/ml) based on a range (different dilutions) of pre‐programmed reference curves for each amino acid. The optimal dilution of nectar: water required for this HPLC method was 1:65, requiring at least 0.25 μl of raw nectar (to make 16.25 μl of solution). The optimal concentration range for our detector was around 10 nM, but we could reliably measure concentration across a range from 0.1 to 1,000 nM. Advanced HPLC grade water was used throughout the study.

#### 2.2.4 Derivation of values

After each compound was identified in each chromatogram, the values produced by the Chromeleon software were scaled up to their original concentrations in nectar based on how much the nectar was diluted (Chromeleon reported values in mol/L). This was carried out in different ways depending on the method used to extract the nectar (see Data S1).

#### 2.2.5 Statistical analysis

Statistical analysis was performed using spss (version 23, IBM Corporation, Armonk, NY, USA). The amino acids were grouped for analysis: essential amino acids (as defined for honeybees, De Groot, 1953: arginine, threonine, phenylalanine, isoleucine, leucine, lysine, methionine, valine, histidine) and non‐essential amino acids (proline, aspartic acid, alanine, cysteine, glutamic acid, glycine, serine, tyrosine and GABA). Tryptophan and glutamine were omitted because they were not at detectable levels in the chromatogram. Glutamic acid and proline were analysed separately, because they were orders of magnitude greater than the rest. Total amino acid concentrations and proline were natural log‐transformed prior to analysis. Total amino acids were analysed in a generalized linear model with method as a main effect. Post hoc analyses were performed using Sidak's test for pairwise comparisons. To examine differences in the profile of amino acids, we performed a principal components method of factor analysis with a Varimax rotation on the data for all amino acids for all five nectar collection methods. The factor scores produced by the analysis were entered into a one‐way generalized linear model (GLM) with nectar collection method as a main effect. Sidak's pairwise post hoc comparisons were made for every method against the microcapillary method.

## 3 RESULTS

**Figure 1:**
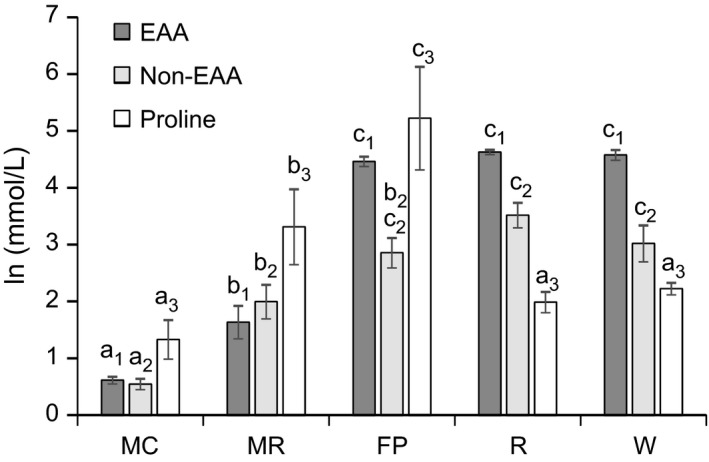
The natural log of the mean (±*SE*) of the total essential amino acid (EAA), non‐essential amino acid (non‐EAA) and proline concentration in nectar samples collected by five methods: MC, microcapillary; FP, filter paper; MR, micro‐rinse; R, rinse 2 ml; and W, wash 2 ml. Letters indicate significant differences (Sidak post hoc tests, *p* < .05) from the microcapillary treatment only. Subscripts indicate specific sets of comparisons; that is, “1” indicates comparisons of EAA across sample collection types, “2” indicates comparisons of non‐EAA, and “3” indicates comparisons of proline

We assume that the microcapillary tube method provides the best representation of what is in nectar; for this reason, we compared all other methods to the data obtained from microcapillary tubes. When microcapillary tubes were used to sample *C. vulgaris* nectar, we found that free amino acids were present at concentrations that were *c*. 880 ± 138 μM for total essential amino acids (EAAs) and *c*. 800 ± 177 μM for total non‐EAAs (excluding proline, Figure 1, Table 2). Proline was present at an order of magnitude greater concentration of 5410 ± 162 μM.

**Table 2:** Mean concentrations (±*SE*) of total essential amino acids (EAAs), total non‐EAAs and proline rendered from each sampling method (MC, microcapillary; MR, micro‐rinse; FP, filter paper; R, rinse 2 ml; and W, wash 2 ml). Units are in mM

Method	Total EAA	Total non‐EAA	Proline
MC	0.883 + 0.138	0.805 ± 0.177	5.41 ± 1.62
MR	7.22 + 2.37	10.8 ± 3.67	158 ± 68.2
FP	89.4 + 9.88	24.8 ± 7.00	812 ± 159
R	101 + 4.98	38.9 ± 5.60	7.17 ± 0.82
W	100 + 8.50	29.6 ± 6.76	8.88 ± 1.27

The method of sampling exerted a strong effect on the relative proportions of the concentrations of total EAA, total non‐EAA and proline in the samples (Figure 1, EAA: GLM, $\chi_{4}^{2}$ = 726, *p* < .001; non‐EAA: GLM, $\chi_{4}^{2}$ = 90.3, *p* < .001; proline: GLM, $\chi_{4}^{2}$ = 35.1, *p* < .001). The total concentration of EAA, non‐EAA and proline was lowest in the microcapillary samples (Figure 1, Table 2). The micro‐rinse and filter paper samples exhibited significantly greater concentrations of EAA, non‐EAA and proline than the microcapillary samples per unit volume. The micro‐rinse method produced values that were *c*. 10‐fold greater than the microcapillary method for EAAs and non‐EAAs and *c*. 30‐fold greater for proline. The filter paper method produced values that were *c*. 100‐fold greater for EAAs, 31‐fold greater for non‐EAAs and 150‐fold greater for proline. The rinse 2 ml and wash 2 ml methods had concentrations of EAAs that were over 100‐fold greater and non‐EAAs that were *c*. fourfold greater than the microcapillary method, but these methods did not significantly overestimate proline (Table 2).

**Figure 2:**
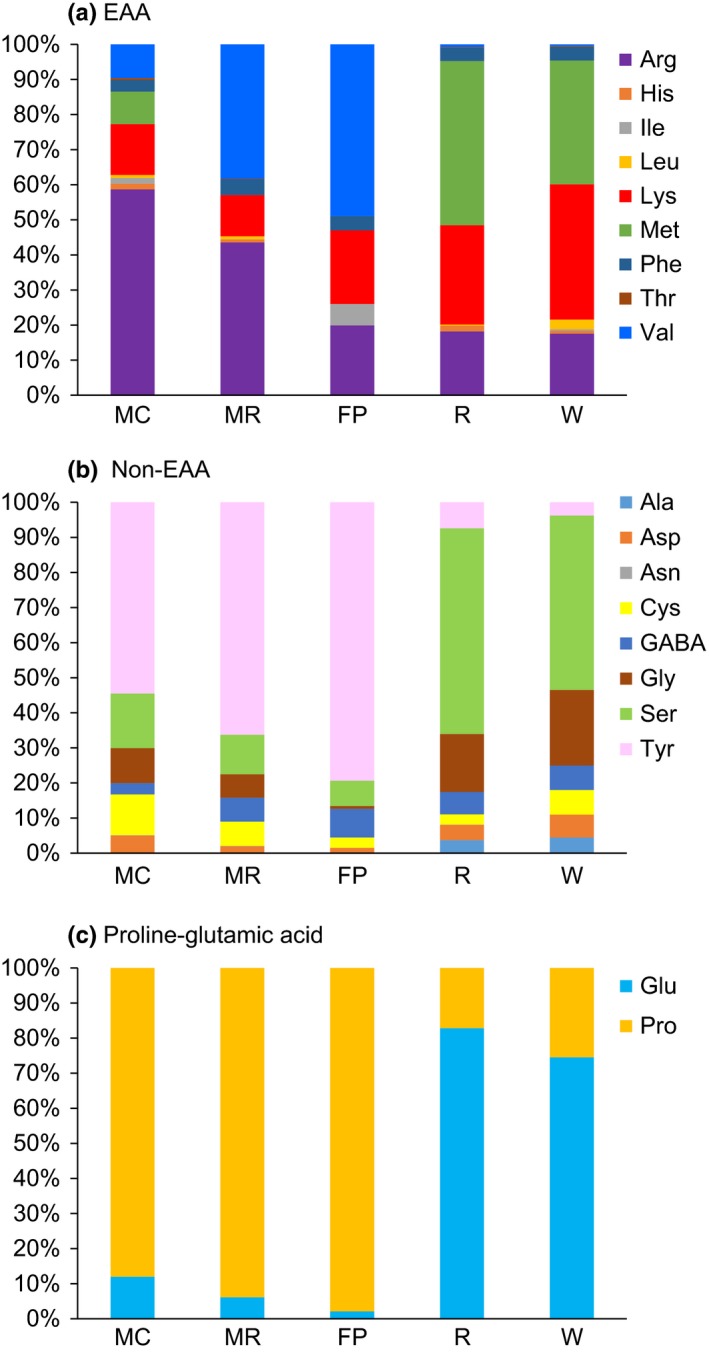
Sampling method affected the proportions of amino acids found in samples from *Calluna vulgaris*. (a) The mean percentage contribution of the essential amino acids (EAA) to nectar samples collected by five methods (excluding tryptophan). (b) The mean non‐essential amino acids (non‐EAAs). (c) The mean percentage of proline and glutamic acid. These amino acids were plotted separately because they were orders of magnitude greater in concentration than all the others. MC, microcapillary; FP, filter paper; MR, micro‐rinse; R, rinse 2 ml and W, wash 2 ml

**Figure 3:**
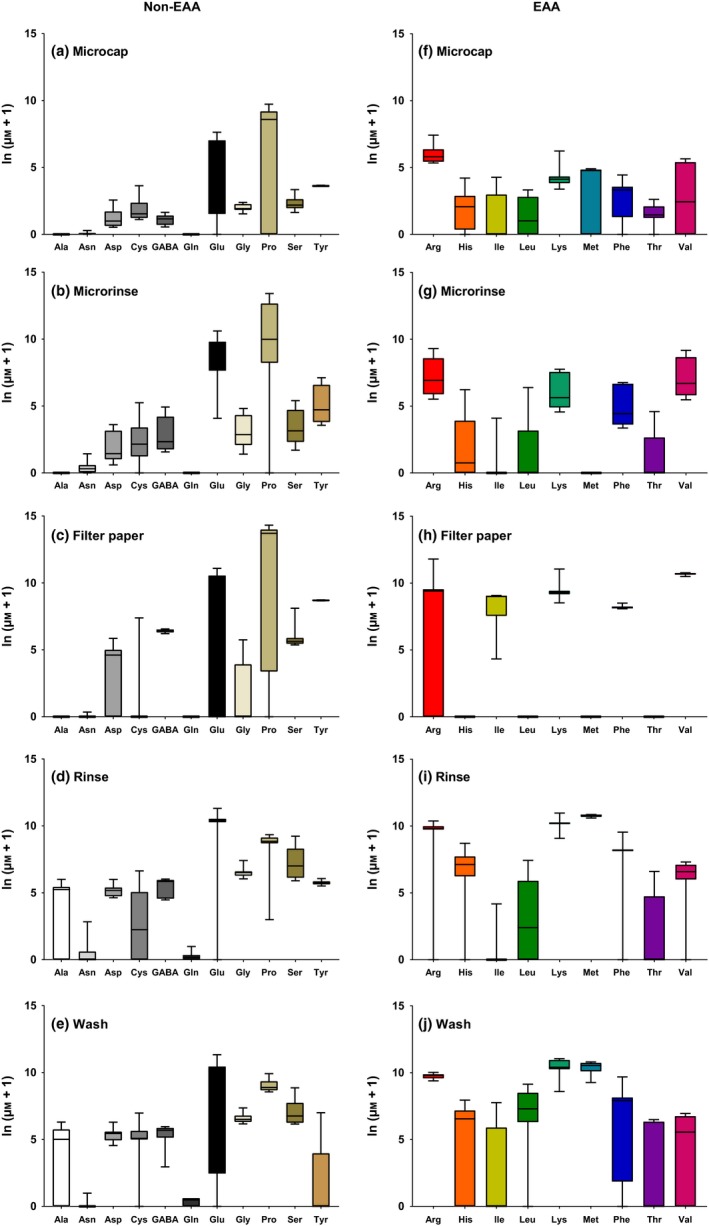
Box‐and‐whisker plots of each amino acid found in the samples from each sampling method. (a–e) Non‐essential amino acids, (f–j) essential amino acids. *N* = 12 samples/method

We also examined how nectar sampling method influenced the relative concentrations of each of the individual amino acids using factor analysis (Figures 2 and 3, Table 3). The amino acids were significantly represented by one of six principal components (F1—6) which accounted for 83% of the variation within the data (Table 3). Most amino acids were significantly positively correlated with the first four factors with the exception of asparagine (F5) and leucine (F6). By analysing the factor scores generated from the factor analysis, we compared how the proportions of the amino acids in the samples were affected by the sampling method. Sampling method significantly influenced the amino acid profiles represented in the factor analysis for F1, F2 and F6 but not the amino acids represented in F3–5 (Table 3). Importantly, the microcapillary and the micro‐rinse methods did not differ significantly in their amino acid profiles (Table 3, Figures 2 and 3). However, the filter paper, rinse 2 ml and wash 2 ml treatments had significantly different amino acid profiles compared to the microcapillary method (Table 3, Figure S2). These differences in the amino acid profiles are especially apparent when the profiles are plotted as percentages of the total amino acids (Figure S2), total EAAs (Figure 2a), total non‐EAAs (Figure 2b) and as the proportion of glutamic acid and proline (Figure 2c—note these were plotted separately because they are a large proportion of the total AAs).

**Table 3:** Factor analysis of amino acids. Top panel: eigenvalues and percentage variance for six factors (F1—6) extracted from all data and the rotated factor matrix. Bold values indicate the factor that best represents each amino acid (i.e. correlation coefficient). Middle panel: one‐way generalized linear model (GLM) comparing methods. Bottom panel: Sidak post hoc pairwise comparisons of each method against the microcapillary method for each factor

	Factor					
	1	2	3	4	5	6
Eigenvalue	5.96	4.38	1.74	1.48	1.14	1.06
% variance	23.4	20.6	15.4	10.5	6.71	6.17
Amino acids						
Alanine (Ala)	−0.213	**0.666**	0.227	0.099	−0.229	−0.134
Arginine (Arg)	0.075	0.164	0.16	**0.904**	0.114	−0.095
Asparagine (Asn)	−0.066	0.027	0.157	0.032	**0.929**	−0.071
Aspartic acid (Asp)	0.059	**0.685**	0.273	0.096	0.007	0.312
Cystine (Cys)	0.111	0.096	0.025	**0.874**	−0.099	0.311
GABA	**0.871**	0.394	−0.029	0.145	−0.03	−0.102
Glutamic acid (Glu)	−0.001	**0.513**	0.39	0.45	0.02	−0.389
Glycine (Gly)	−0.215	**0.778**	0.21	0.211	−0.001	0.337
Histidine (His)	−0.156	0.261	**0.755**	0.003	0.384	0.083
Isoleucine (Ile)	**0.891**	−0.081	−0.057	−0.077	−0.037	0.074
Leucine (leu)	−0.112	0.305	0.232	0.149	−0.066	**0.737**
Lysine (lys)	0.148	**0.854**	0.019	0.009	0.095	0.164
Methionine (Met)	−0.255	**0.872**	0.193	0.094	0.141	−0.067
Phenylalanine (Phe)	0.28	0.428	**0.596**	0.106	−0.307	−0.182
Proline (Pro)	**0.859**	−0.17	−0.059	−0.09	−0.014	0.029
Serine (Ser)	−0.076	0.188	**0.879**	0.203	−0.108	0.11
Threonine (Thr)	−0.114	0.121	**0.877**	0.023	0.213	0.142
Tyrosine (Tyr)	**0.953**	−0.111	−0.035	0.147	−0.039	−0.107
Valine (Val)	**0.956**	−0.152	−0.052	0.153	−0.036	−0.079
1‐way GLM						
$\chi_{4}^{2}$	597	186	6.97	3,23	5.07	19.6
*p*	**<.001**	**<.001**	.137	.520	.281	**<.001**
*Sidak's pairwise* post hoc tests against the microcapillary method						
*p*‐value						
Filter paper	.419	.931	.925	.985	.990	.969
Micro‐rinse	**<.001**	**.003**	.925	.733	.990	.969
Rinse 2 ml	**.047**	**<.001**	.231	.925	.441	.548
Wash 2 ml	**.013**	**<.001**	.679	.852	.990	**.050**

Note:

Post hoc comparisons are restricted to each column.

GABA, gamma‐aminobutyric acid.

Proline was the last amino acid off of the column and hence occurred at the end of the UHPLC run (Figure S1). For this reason, the potential for contamination of this peak (from non‐amino acid solutes in nectar) was considerably greater than for other amino acids. In general, the concentration of proline was greater than any other amino acid (Figure 1). The most proline was found in the filter paper method samples compared to any of the other methods. To identify whether the filter paper method introduced amino acid contamination, we also performed a simple rinse of the filter paper and analysed this on the UHPLC. We found that the filter paper added very low amounts (0.1% of nectar concentration) of 15 of the amino acids we measured (Table S1), but not a specific, larger spike in proline.

## 4 DISCUSSION

The method used to collect nectar considerably influenced the mean amino acid concentrations in nectar from *C. vulgaris*. Samples obtained by the wash 2 ml, rinse 2 ml and filter paper methods contained much higher amino acid concentrations than the microcapillary and micro‐rinse methods. The amino acid profiles were also affected by the sampling methods such that only the micro‐rinse method yielded a profile matching that of the microcapillary method.

The main source of amino acid contamination found in the filter paper, rinse 2 ml and wash 2 ml methods is likely to be from floral pollen (Gottsberger et al., 1990). Contamination of the microcapillary samples with pollen amino acids happens less frequently because the microcapillary tubes are narrow and can be positioned directly around the nectary. However, the microcapillary method is not always effective in extracting nectar from flowers with low‐nectar volumes because the nectar around the nectaries may be too viscous to be removed by capillary action or there may not be enough nectar to extract. Of all the methods in our study, the micro‐rinse method returned the most similar results to the microcapillary method. The micro‐rinse method samples exhibited a similar amino acid profile but a greater total amino acid content than the microcapillary samples. We believe that this difference in the measurement of total amino acids occurred because of the dilution factor used to back‐calculate the concentration for the micro‐rinse method. This dilution factor affects the magnitude of the total amount of each amino acid and inaccuracies can arise because of difficulties in recovering the nectar and all of the 2 μl injected into each flower. For this reason, when using the micro‐rinse method, it is important to try to obtain a few samples using microcapillary tubes to estimate the volume of the standing crop of nectar in each flower. Despite the care that must be taken with the micro‐rinse method, our results indicate that it is less likely to produce samples that are contaminated with amino acids from pollen than the other methods described here. For this reason, we conclude that the micro‐rinse method is the best alternative method to microcapillary tubes for approximating concentrations of amino acids found in nectar of small‐volume flowers.

Other studies previously used filter paper to extract nectar for amino acid analysis of nectar (McKenna & Thomson, 1988; Petanidou et al., 2006). The filter paper method in our study, however, produced samples with significantly higher concentrations of amino acids (particularly essential amino acids and proline) than the microcapillary method; the amino acid composition was also significantly different. We expect that filter paper works less well for amino acid measurements because it is very difficult to prevent it being contaminated with pollen when inserted into small flowers. Therefore, we do not recommend using the filter paper method to analyse amino acids because of the risk of contamination.

Like the filter paper method, the wash 2 ml and rinse 2 ml methods recovered significantly higher total amino acids and different amino acid profiles than the microcapillary method. This could have occurred for at least two reasons. First, using these methods, it is difficult or impossible to exclude free amino acids from pollen in these samples. Removing anthers with tweezers prior to sampling runs the risk of vascular fluid leaking into the sample. Nectar collected from flowers damaged in this way were found to have altered amino acid profiles (Gottsberger et al., 1990). Amino acid concentrations in phloem were measured as 121–300 mM for plants like alfalfa and spinach, and cytosolic concentration was 121 mM (Girousse, Bournoville, & Bonnemain, 1996; Riens, Lohaus, Heineke, & Heldt, 1991). These values are *c*. 500–1,000 times more concentrated than the amino acids found in our nectar samples. Lohaus and Schwerdtfeger (2014) found the nectars of *Maurandya barclayana, Lophospermum erubescens* and *Brassica napus* to have much lower amino acid concentrations than their respective phloems. Sealing the cut surface with wax or surgical glue may prevent fluid leakage (Morrant et al., 2009) but would be extremely time‐consuming and difficult to accomplish with tiny flowers.

Alternatively, washing or rinsing with large volumes of fluid could elute dried nectar solutes into the sample. Using this method, however, it would be necessary to dry down each sample and reconstitute it in water. The actual concentration found in the nectar would remain unknown, but the total amount of each solute available could be calculated. However, this might not be what an animal foraging for nectar could acquire from a flower. For example, in a study of methods for carbohydrate analysis of nectar, Morrant et al. (2009) and Petit, Rubbo, and Schumann (2011) found that nectar collected using microcapillaries contained lower quantities of carbohydrates than nectar collected using rinse or wash methods. We suspect that this is because washing dissolves carbohydrates that have dried on the inner petal surfaces. These dried nectar constituents are unlikely to be available to most floral visitors (except for insects like flies that can use salivary secretions to take up substrates). Bees, however, do not use salivary secretions to recover food and spend very little time probing each flower for nectar. For example, bumblebees spent between 0.5 and 3 s per flower on a variety of plant species (time spent foraging was correlated with corolla length) (Inouye, 1980). Two bumblebee species' nectar removal rates were between 0.3 and 0.4 μl/s in two high‐nectar producing plant species (Graham & Jones, 1996). For this reason, rapid licking or sucking near the nectary is unlikely to involve much ingestion of solutes present in crystallized form across the entire flower surface. Our data indicate that washing or rinsing the flowers with high volumes of solvent also alters the profile of amino acids recovered during sampling and might not represent what a pollinator would obtain from the flower when collecting nectar. For these reasons, caution is required if using the wash 2 ml or rinse 2 ml methods for nectar sampling for amino acid analysis.

When analysing nectar for amino acids, it is important to reduce contamination of samples because free amino acids are widespread in the environment and are present in very low concentrations in nectar. In addition to contamination of sample from equipment/solvent contact with other floral parts, contamination from unsterilized laboratory equipment, chemicals, solvents, hands/skin and paper is also a major concern. We used a specific brand of de‐ionized water because we found that distilled water and some brands (including laboratory filtration systems) of de‐ionized water were contaminated with amino acids, particularly cystine and glutamine. We found that proline values for some samples were very high and erratic, particularly in the filter paper method, perhaps because of co‐elution of other contaminants from the filter paper with proline, elevating the estimated level of proline because these compounds co‐elute at the end of the run and are detected by our fluorescence detector.

There are drawbacks associated with different nectar collection methods because their effectiveness is influenced by floral morphology, nectar characteristics, sampling regime (Bolton, Feinsinger, Baker, & Baker, 1979; Kearns & Inouye, 1993; Lloyd, Ayre & Whelan, 2002; Morrant et al., 2009), nectar volume and the intended chemical analysis. It is clear from our study using *C. vulgaris* that nectar amino acid recovery from small‐volume flowers depends on collection method. Our experiments show that the micro‐rinse method delivers the closest estimate to the microcapillary tube method of nectar extraction. In conclusion, we recommend that, if the intended chemical analysis involves only carbohydrates, then the use of microcapillaries, a micro‐rinse or filter paper will suffice, but if the intended analysis is to include other solutes such as amino acids then only the microcapillary or the micro‐rinse methods are reliable ways of identifying the amino acid profile. Furthermore, testing samples for the presence of pollen (e.g. by microscopic verification) is also a means of ruling out whether or not pollen contamination affects the amino acid profile.

## AUTHOR CONTRIBUTIONS

E.F.P., D.S. and G.A.W. designed the experiments; E.F.P., D.S., G.A.W., A.M.B. and J.B. edited and wrote the manuscript; D.S. collected the data; E.F.P. and G.A.W. analysed the data.

## DATA ACCESSIBILITY

The data from these experiments can be found at figshare.com https://doi.org/10.6084/m9.figshare.5532757.
