# Supplementary material for: Analysis of nectar from low‐volume flowers: A comparison of collection methods for free amino acids
Source: Methods Ecol Evol. 2017 Nov 14;9(3):734–43. doi: 10.1111/2041-210X.12928 (PMC5993345; doi:10.1111/2041-210X.12928)
Supplement: Supplementary file 1 [file MEE3-9-734-s001.docx]

Supplementary Information:

**Methods:**

Dilution factors

This section explains how we estimated the concentration of amino acids in raw nectar by taking into account how much the nectar was diluted in the flower (using the micro-rinse method) and how much it was subsequently diluted for UHPLC analysis.

Microcapillary method: the raw nectar extracted directly from the flower was diluted to make enough sample for the chromatography instruments. Therefore, we multiplied the measured amino acid concentrations by the dilution factor used to dilute the raw nectar before analysis (65 fold for our instruments). The filter paper, wash and rinse methods yielded volumes of 2000 µl. To estimate how much the raw nectar in the flower was diluted we divided the total amount of water added to extract the nectar (2000 µl) by the mean standing crop per flower. The standing crop was used as an estimate of how much nectar was in the flower at the time of sampling. The mean standing crop per flower (n=12) recovered using microcapillary tubes was 0.474 µl (± 0.06 SE). See equation 1:

$$D = \frac{W}{S}$$

Where: D = final dilution factor; W = amount of water (µl) added to flower during nectar sampling; S = standing crop (µl). The final dilution factor was multiplied by the Chromeleon sample measurement (mol/L) for each amino acid to obtain approximate raw nectar values.

The micro-rinse method: raw nectar was first diluted by adding 2 µl to the flower to extract the nectar. On average a volume of 2.005 µl (+ 0.113 SE) (water and nectar) was recovered from each flower. This water/nectar solution (aka recovery volume) was brought up to a final volume of 30 µl by adding purified water (to provide enough sample for analysis in the UHPLC machine). The volume of raw nectar in each flower was estimated by subtracting the mean recovery volume (2.005 µl + 0.113) from the mean standing crop (0.474 µl; see above measured by the microcapillary method). This value, 1.53 µl, was our estimate of the water recovered from the micro-rinse method. We subtracted this estimate from the actual recovery volume per sample to calculate the estimate of the nectar volume. Step 1: To calculate the dilution factors, the recovery volume from each flower was divided by the mean standing crop. This represented the dilution that occurred when 2 µl was added to the flower. Step 2: The recovery volume was brought up to volume to 30 µl (specific to our UHPLC machine sample size requirements). To calculate the dilution factor for this step, we divided 30/(recovery volume). The dilution factors from step 1 and step 2 were multiplied with the concentration measured in the sample by the HPLC machine to calculate the raw nectar values.

**Supplemental figures:**

**
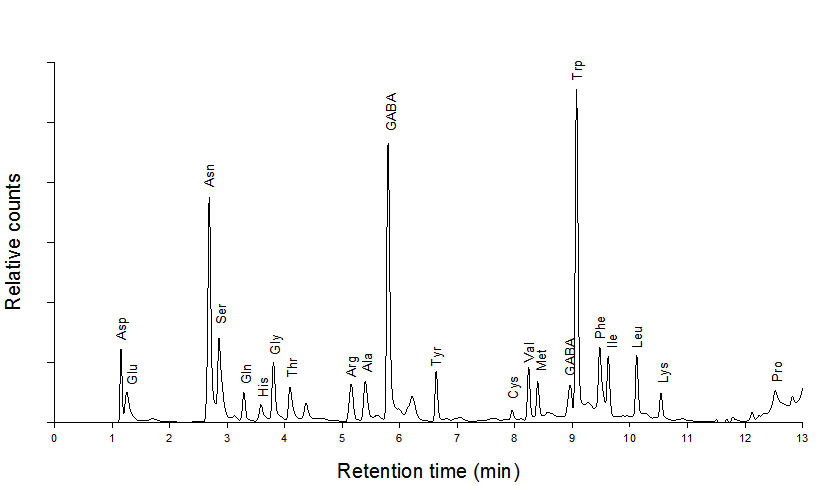
**

Figure S1: Example chromatogram showing retention times for 21 amino acids quantified. Note that GABA elutes as two peaks.

Figure S2: The mean percentage contribution of all amino acids (excluding proline and glutamic acid) to nectar samples collected by five methods: MC = microcapillary; MR = micro-rinse; FP = filter paper; R = rinse 2ml and W = wash 2ml.

Table S1: Mean amino acid concentration in samples from five nectar collection methods and a filter paper (FP) control (units in mmol/l). MC = microcapillary; MR = micro-rinse; FP = filter paper; R = rinse 2ml and W = wash 2ml.

|  |  |  |  |  |  |
| --- | --- | --- | --- | --- | --- |

| **Amino Acid** | **MC** | **MR** | **FP** | **R** | **W** | **FPcontrol** |
| --- | --- | --- | --- | --- | --- | --- |
| Ala | <0.001 | <0.001 | <0.001 | 0.160 | 0.161 | 0.01 |
| Arg | 0.518 | 3.15 | 17.8 | 18.6 | 17.5 | 0.01 |
| Asn | <0.001 | 0.0006 | 0.0001 | 0.002 | 0.0002 | <0.001 |
| Asp | 0.003 | 0.011 | 0.115 | 0.189 | 0.233 | <0.001 |
| Cys | 0.008 | 0.037 | 0.224 | 0.125 | 0.252 | 0.01 |
| GABA | 0.002 | 0.037 | 0.616 | 0.277 | 0.251 | 0.01 |
| Glu | 0.738 | 10.4 | 17.3 | 34.7 | 26.0 | 0.01 |
| Gly | 0.007 | 0.036 | 0.059 | 0.711 | 0.772 | 0.01 |
| His | 0.015 | 0.058 | <0.001 | 1.64 | 0.806 | 0.01 |
| Ile | 0.014 | 0.006 | 5.46 | 0.005 | 0.462 | 0.01 |
| Leu | 0.008 | 0.063 | <0.001 | 0.331 | 2.78 | 0.01 |
| Lys | 0.128 | 0.849 | 18.7 | 28.9 | 38.6 | 0.01 |
| Met | 0.082 | <0.001 | <0.001 | 47.7 | 35.3 | <0.001 |
| Phe | 0.029 | 0.323 | 3.65 | 3.89 | 4.00 | 0.01 |
| Pro | 5.41 | 158 | 812 | 7.18 | 8.88 | 0.01 |
| Ser | 0.010 | 0.061 | 0.546 | 2.52 | 1.78 | 0.01 |
| Thr | 0.005 | 0.012 | <0.001 | 0.161 | 0.200 | <0.001 |
| TYR | 0.036 | 0.357 | 5.98 | 0.318 | 0.135 | <0.001 |
| Val | 0.085 | 2.77 | 43.8 | 0.771 | 0.400 | 0.010 |
